# Viral miRNAs in plasma and urine divulge JC polyomavirus infection

**DOI:** 10.1186/1743-422X-11-158

**Published:** 2014-09-02

**Authors:** Ole Lagatie, Tom Van Loy, Luc Tritsmans, Lieven J Stuyver

**Affiliations:** Janssen Diagnostics, Turnhoutseweg 30, Beerse, 2340 Belgium; Janssen Research and Development, Turnhoutseweg 30, Beerse, 2340 Belgium

**Keywords:** JC Polyomavirus, Viral microRNA, Circulating microRNA, Progressive Multifocal Leukoencephalopathy, Biomarker, Viral activity

## Abstract

**Background:**

JC polyomavirus (JCPyV) is a widespread human polyomavirus that usually resides latently in its host, but can be reactivated under immune-compromised conditions potentially causing Progressive Multifocal Leukoencephalopathy (PML). JCPyV encodes its own microRNA, jcv-miR-J1.

**Methods:**

We have investigated in 50 healthy subjects whether jcv-miR-J1-5p (and its variant jcv-miR-J1a-5p) can be detected in plasma or urine.

**Results:**

We found that the overall detection rate of JCPyV miRNA was 74% (37/50) in plasma and 62% (31/50) in urine. Subjects were further categorized based on JCPyV VP1 serology status and viral shedding. In seronegative subjects, JCPyV miRNA was found in 86% (12/14) and 57% (8/14) of plasma and urine samples, respectively. In seropositive subjects, the detection rate was 69% (25/36) and 64% (23/36) for plasma and urine, respectively. Furthermore, in seropositive subjects shedding virus in urine, higher levels of urinary viral miRNAs were observed, compared to non-shedding seropositive subjects (*P* < 0.001). No correlation was observed between urinary and plasma miRNAs.

**Conclusion:**

These data indicate that analysis of circulating viral miRNAs divulge the presence of latent JCPyV infection allowing further stratification of seropositive individuals. Also, our data indicate higher infection rates than would be expected from serology alone.

**Electronic supplementary material:**

The online version of this article (doi:10.1186/1743-422X-11-158) contains supplementary material, which is available to authorized users.

## Introduction

The human JC polyomavirus (JCPyV) is the etiological agent of Progressive Multifocal Leukoencephalopathy (PML), a demyelinating disease of the brain caused by lytic infection of oligodendrocytes upon viral reactivation [1]. JCPyV is a circular double-stranded DNA virus with very restricted cellular tropism, infecting oligodendrocytes, astrocytes, kidney epithelial cells and peripheral blood cells [2, 3]. It is thought that infection usually occurs asymptomatically in childhood, after which the virus remains latent in the body [4–6]. Under certain immunocompromising conditions, such as treatment with immunomodulatory drugs (e.g. natalizumab) or infection with Human Immunodeficiency Virus (HIV), the virus can be reactivated and actively replicate into the brain, leading to PML. Current risk assessment for development of PML is mainly based on the detection of antibodies against VP1, the major capsid protein and the detection of viral DNA in urine (viruria). It has been reported that 50 to 80% of humans are seropositive for JCPyV and approximately one fifth of the population sheds JCPyV in the urine [7–13]. Detection of viral DNA in plasma (viremia) is very rare and has been shown not to be useful for predicting PML risk [14–16]. Recently it was shown, however, that viral DNA can be detected in CD34^+^ or CD19^+^ cells, with an increased detection rate in Multiple Sclerosis (MS) patients treated with natalizumab [3]. As the risk of developing PML increases upon prolonged use of natalizumab, current treatment guidelines recommend discontinuation of therapy after the second year, particularly in JCPyV seropositive patients [17]. Given the high prevalence of JCPyV antibodies, a large number of patients are advised to discontinue therapy. Although most, if not all, PML patients are seropositive or show seroconversion before diagnosis of PML, the incidence of PML in natalizumab-treated MS patients is not more than 1.1% in the highest risk subgroup, indicating that not all seropositive subjects have the same risk of developing PML [18]. Moreover, the introduction of a risk stratification algorithm, predominantly based on JCPyV serology, has not led to a reduction of PML incidence in natalizumab-treated MS patients [19]. Therefore, development of new tools for improved risk stratification is warranted, as this might justify continued therapy for many MS patients and better identify those patients who are really at risk of developing PML.

MicroRNAs (miRNAs) are small, non-coding RNAs that play an important role in fine-tuning the expression of specific gene products through translational repression and/or mRNA degradation and as such are implicated in many diseases [20–22]. Cellular miRNAs can also be released in small vesicles, such as exosomes and the levels of these extracellular miRNAs in biological fluids have become very valuable markers of several diseases, such as cancer, Alzheimer’s disease and diabetes [23–25]. In the context of JCPyV, it was shown that there does not appear to be a relationship between circulating human miRNAs and the presence of anti-VP1 antibodies or urinary viral load [26].

Several viruses encode their own sets of miRNAs, which can have self-regulatory or host modulating roles [22]. Also JCPyV, as well as other polyomaviruses, encodes its own unique microRNA that is produced as part of the late transcript in an infected host cell [27, 28]. These microRNAs are thought to play an important role in controlling viral replication through downregulation of Large T-Antigen expression, but also in controlling NKG2D-mediated killing of virus-infected cells by natural killer (NK) cells through downregulation of the stress-induced ligand ULBP3 [28, 29].

The diagnostic potential of circulating viral miRNAs has already been investigated for Epstein-Barr virus and Kaposi’s sarcoma-associated herpesvirus (KSHV), where they might represent potential markers for virus associated malignancies [30, 31]. Also JCPyV miRNA has been shown recently to be a useful biomarker for JCPyV infection in the gastrointestinal tract [32]. In this study we have investigated whether JCPyV-encoded miRNAs could be detected in plasma or urine of healthy subjects and whether the presence of these miRNAs was related to VP1 serology or urinary viral load. We demonstrate that these viral miRNAs can indeed be detected in plasma or urine and that they might be useful markers for JCPyV infection.

## Results

### Assay linearity and specificity

**Figure 1:**
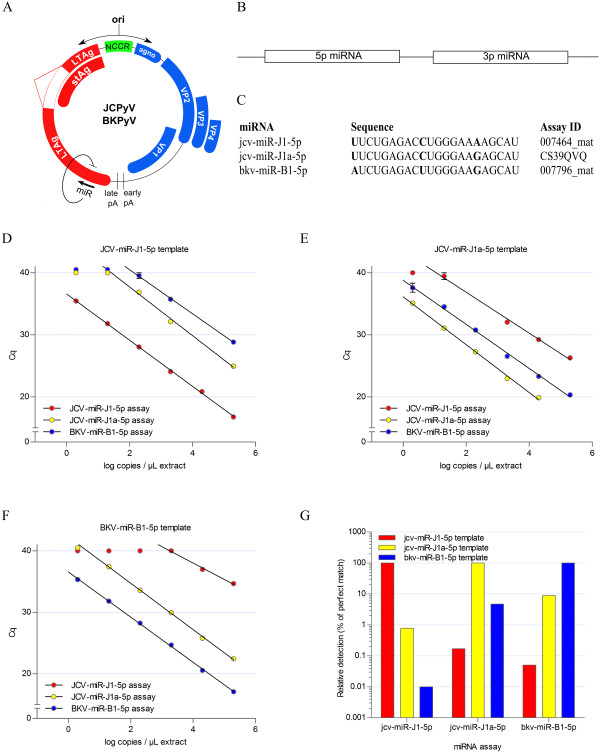
**Validation of the stem-loop RT-PCR miRNA assays.** **(A)** Genomic location of the JCPyV/BKPyV encoded miRNAs. **(B)** Graphical representation of a pre-miRNA as precursor of the mature 5p and 3p miRNAs generated upon cleavage by Dicer **(C)** Sequence comparison of the three miRNAs investigated. **(D-F)** The assay linearity and specificity was evaluated with dilution series of three synthetic miRNAs, miR-J1-5p, miR-J1a-5p and miR-B1-5p. Each dilution series was analyzed using the three miRNA assays. **(G)** The assay readings of miR-J1-5p by miR-J1-5p assay, miR-J1a-5p by miR-J1a-5p assay, and miR-B1-5p by miR-B1-5p assay at concentration levels of 2.10^4^ copies/μL extract were used as the relative standards (100%) for the analysis of assay cross-reactivity.

Plasma or urine levels of JCPyV miRNA were analyzed using stem–loop RT followed by TaqMan PCR analysis [33]. Since the 3p miRNA of JCPyV is identical to the 3p miRNA of BKPyV, only 5p miRNAs were investigated [28]. As we previously identified a variant of the JCV-miR-J1-5p bearing one nucleotide difference compared to the miRNA described in miRBase (designated JCV-miR-J1a-5p), assays specifically designed for both variants were used, as well as an assay detecting the closely related BKPyV 5p miRNA (Figure 1A-C) [26]. To evaluate the specificity of the assays, standard curves were prepared of each miRNA (JCV-miR-J1-5p, JCV-miR-J1a-5p and BKV-miR-B1-5p) and analyzed using the three specific assays (Figure 1D-F). Relative detection efficiency was calculated from the difference of quantification cycle (Cq) between the specific assay and the non-specific assay, using samples containing 2.10^4^ copies/μL of the individual miRNAs (Figure 1G). Only marginal cross reaction was observed for most combinations, but a substantial cross reaction was observed between JCV-miR-J1a-5p and BKV-miR-B1-5p. Therefore, for every single clinical sample (plasma or urine), the contribution of non-specific amplification was calculated based on these standard curves. For JCV-miR-J1-5p and JCV-miR-J1a-5p it was confirmed that the contribution of non-specific signal was negligible compared to the specific signal (Additional file 1: Table S1 and Additional file 2: Figure S1). For the BKV-miR-B1-5p assay, however, the calculated contribution of JCV-miR-J1a-5p in most cases was similar to the measured levels, indicating that the BKV-miR-B1-5p assay in most cases actually was detecting JCV-miR-J1a-5p. Consequently, we can conclude that in most samples BKV-miR-B1-5p is absent or present at such low levels that it does not interfere in the interpretation of the JCV-miR-J1-5p and JCV-miR-J1a-5p analyses.

### Analysis of JCPyV miRNA levels in plasma and urine

Plasma and urine samples were collected from 50 healthy subjects (HS) and JCPyV VP1 serology and both plasma and urinary viral load was determined (Additional file 3: Table S2). Based on these parameters, subjects were categorized as Ab^-^, Ab^+^VL^-^ or Ab^+^VL^+^ (Table 1). Among these 50 HS JCPyV VP1 antibody prevalence was 72% (36/50) and 36% (13/36) of this group were also shedding virus in their urine, representing 26% (13/50) of the total study population. In the JCPyV VP1 seronegative group, no urinary virus shedding was observed. We found no subjects with detectable JCPyV DNA in plasma, similar to previous observations [16].

**Table 1:** **Overview of subjects investigated**

Variable		Healthy subjects (n = 50)
Gender, n (%)		
	Male	22 (44%)
	Female	28 (56%)
	Age, median (Min-Max)	40.5 (23-59)
Race, ethnicity, n (%)		
	White	38 (76%)
	Black	3 (6%)
	Asian	8 (16%)
	Other/unknown	1 (2%)
VP1 serology, n (%)		
	Positive	36 (72%)
	Negative	14 (28%)
Viruria, n (%)		
	Positive*	13 (26%)
	Negative	37 (74%)
Viremia, n (%)		
	Positive	0 (0%)
	Negative	50 (100%)

*All viruric subjects were part of the VP1 seropositive subgroup.

**Figure 2:**
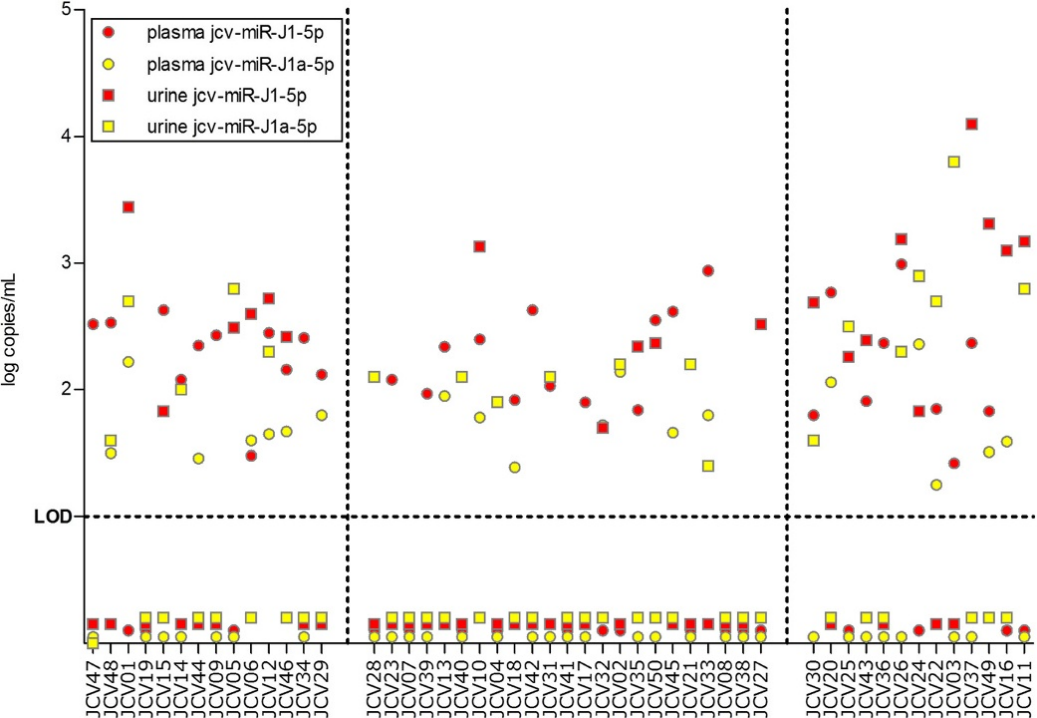
**Individual levels of jcv-miR-J1-5p and jcv-miR-J1a-5p in plasma and urine.** Plasma and urine levels (in log copies/mL) of the two JCPyvV miRNA variants (jcv-miR-J1-5p and jcv-miR-J1a-5p) in every individual subject.

**Figure 3:**
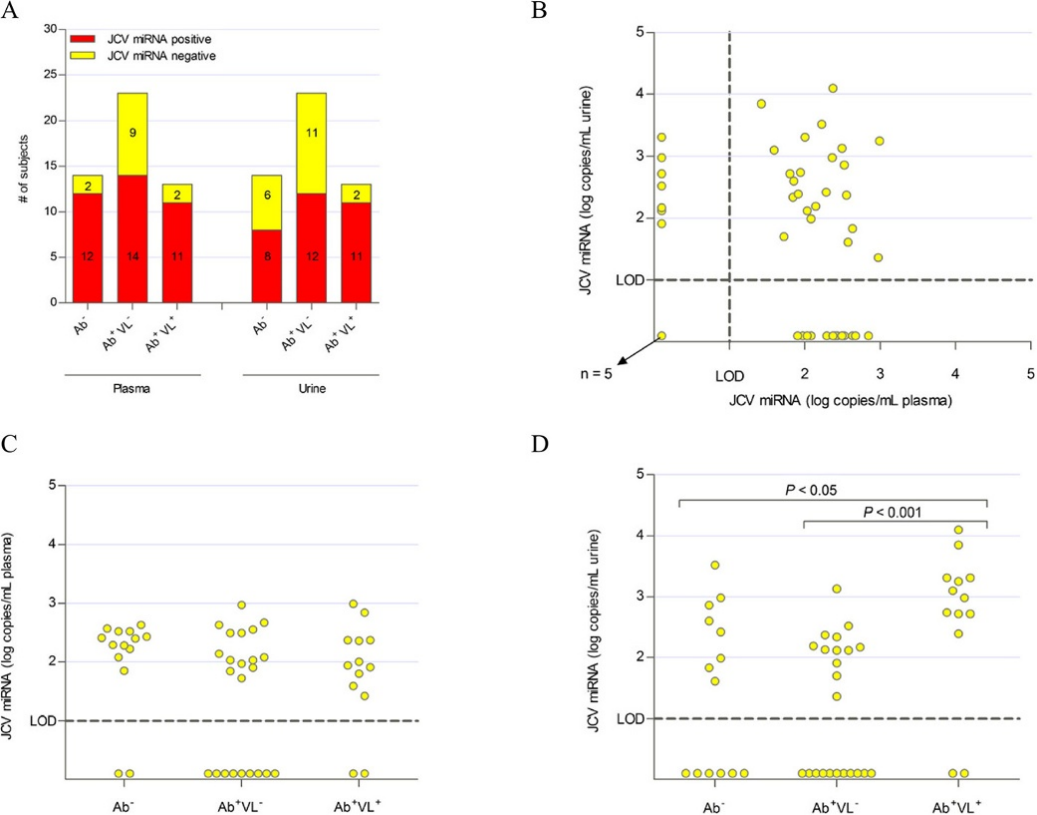
**JCPyV miRNA levels detected in plasma and urine of healthy subjects, categorized based on serology and urinary viral load. (A)** Number of subjects with detectable levels of JCPyV miRNAs in plasma and urine in the different groups. **(B)** Correlation between plasma and urine levels (in log copies/mL) of JCPyV miRNAs. **(C)** Plasma levels (in log copies/mL) of JCPyV miRNAs in the different groups. In case both variants were detected, the sum of both levels is presented. **(D)** Urine levels (in log copies/mL) of JCPyV miRNAs in the different groups. In case both variants were detected, the sum of both levels is presented.

Total RNA, including microRNAs was isolated from both urine and plasma and the level of JCPyV 5p miRNA was quantified in all samples (Figure 2). The overall detection rate of JCPyV miRNA was 74% (37/50) in plasma and 62% (31/50) in urine (Figure 3A). Further analysis of the different subgroups shows that JCPyV miRNA was detected in plasma or urine from HS from all subgroups (Ab^-^, Ab^+^ VL^-^ or Ab^+^VL^+^) at similar detection rates (*P* > 0.05 between the subgroups for both plasma and urine). In the seropositive group, JCPyV 5p miRNA was detected in plasma of 69% (25/36) of subjects and in urine of 64% (23/36) of subjects. Remarkably, also in the seronegative group, JCPyV 5p miRNA was detected in plasma of 86% (12/14) of subjects and in urine of 57% (8/14) of subjects. These detection rates were not statistically different compared to those in seropositive subjects (*P* > 0.05 both for plasma and urine).

**Figure 4:**
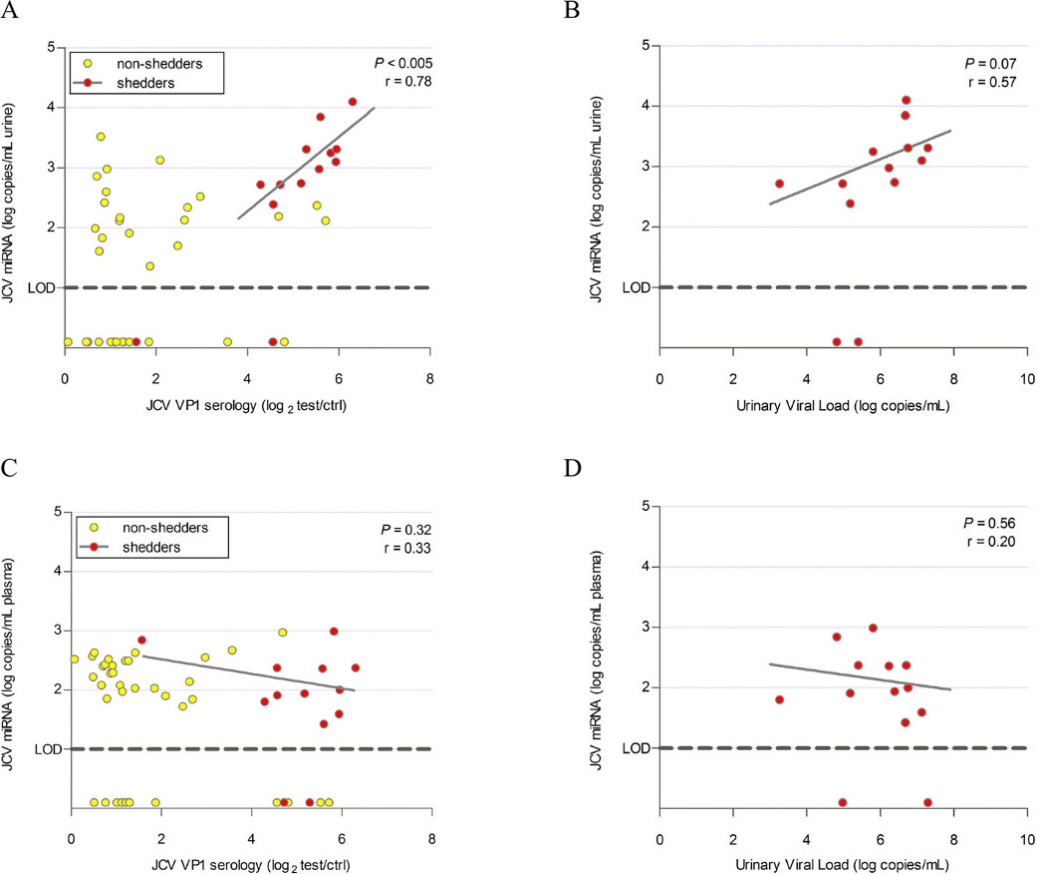
**Viral miRNAs related to other viral parameters. (A)** Correlation between JCPyV miRNA levels (in log copies/mL) in urine and JCPyV VP1 serology (in log_2_ test/ctrl). Shedders are indicated in red and *P*-value and r value are based on this subset only. **(B)** Correlation between JCPyV miRNA levels (in log copies/mL) in urine and JCPyV urinary viral load (in log copies/mL) in shedders. **(C)** Correlation between JCPyV miRNA levels (in log copies/mL) in plasma and JCPyV VP1 serology (in log_2_ test/ctrl). Shedders are indicated in red and *P*-value is based on this subset only. **(D)** Correlation between JCPyV miRNA levels (in log copies/mL) in plasma and JCPyV urinary viral load (in log copies/mL) in shedders.

Quantitative analysis of JCPyV 5p miRNA indicated plasma levels of JCPyV 5p miRNA were similar in the three different subgroups (Figure 3C). In urine, significantly higher levels (*P* < 0.001) were observed in the subgroup shedding JCPyV in their urine compared to the subgroup not shedding JCPyV in their urine (Figure 3D). No correlation was observed between plasma levels and urine levels of JCPyV 5p miRNA (Figure 3B). Remarkably, while in plasma higher levels of JCV-miR-J1-5p were detected than JCV-miR-J1a-5p, in urine both variants were detected at similar levels (Figure 2). Also, the identity of the miRNAs (J1 or J1a variant) was not correlated between urine and plasma. Comparison of JCPyV 5p miRNA levels with JCPyV VP1 serology or urinary viral load showed that, specifically in the subgroup of JCPyV shedders (Ab^+^VL^+^) a good correlation (*P* < 0.005, r = 0.78) exists between miRNA levels and antibody levels, as well as a moderate correlation (*P* = 0.07, r = 0.57) with urinary viral load (Figure 4A-B). No correlation could be observed between plasma miRNA levels and any other parameter analyzed (Figure 4C-D).

## Discussion

Although JCPyV is known to be the etiological agent responsible for development of PML, it is also well-known that this polyomavirus is widely distributed in the human population without any clinical manifestation [1]. In this study we have analyzed the level of the viral miRNAs in plasma and urine of healthy subjects. As is the case for other polyomaviruses, JCPyV encodes a pre-miRNA that is further processed into the mature 5p and 3p miR-J1s. The pre-miRNA is encoded on the late strand of the viral genome and is shown to be expressed concurrently with the late mRNA transcript, thereby downregulating early transcript [27, 28]. The presence of viral miRNAs might therefore be considered as a marker for latent infection. Since BKPyV and JCPyV share the same 3p miRNA, only the 5p miRNAs were included in this study [28].

We showed that most of the healthy subjects have low, but well-detectable levels of viral miRNAs in total RNA extracts of plasma or urine, even in subjects that are seronegative and as such considered not to be infected. This finding indicates that the analysis of antibodies against JCPyV VP1 is insufficient to identify all infected individuals. Previous studies also identified individuals that were seronegative but clearly infected based on the fact that they were viremic in specific cell compartments (CD34^+^ cells) or plasma [3, 34–36]. Whereas in most cases only a limited number of seronegative subjects was found to be infected, our analysis of viral miRNAs in plasma and urine identified, respectively 12 and 8 out of 14 seronegative individuals to be infected with JCPyV. Only 1 seronegative subject was found to be negative for both plasma and urine viral miRNAs. These data suggest that JCPyV is capable of evading immune recognition by the host’s humoral immune system and residing latently in the body, with viral miRNAs leaking in the blood or urine as a silent witness of this latent activity.

Similar to the findings in seronegative subjects, also a large group of seropositive subjects was found to have viral miRNAs in plasma or urine. 69% of seropositive subjects were positive for plasma viral miRNAs and 64% were found to be positive for urinary viral miRNAs. Given the fact that viral miRNAs can only be produced upon ongoing viral transcription in infected host cells, whereas serology rather discloses information on exposure to the virus and more in particular the response of the humoral immune system towards this exposure, the analysis of viral miRNAs in seropositive subjects might add another level to the risk prediction algorithms for PML development. We therefore suggest that further studies would be performed to investigate the potential of viral miRNA levels in plasma or urine as a risk marker for PML development.

Plasma levels of viral miRNAs in seropositive subjects were not different compared to seronegative subjects and both were close to the detection limit, indicating that in healthy subjects only low activity of JCPyV is present in the periphery, independent of their antibody status. In contrast, we found significantly increased urinary viral miRNA levels in shedders. In this group of viral shedders, there was also a strong correlation between urinary miRNA levels and anti-VP1 antibody levels or urinary viral load.

Besides the discordance between urine and plasma miRNA levels, also the identity of the detected miRNAs (J1-5p or J1a-5p variant) within individual subjects was different in plasma and urine. The most obvious explanation for these observations would be that two independent viral propagation zones exist in one individual: one in bone marrow cells, blood cells and perhaps also brain cells (Bone-Blood-Brain) and a second one in urinary tract cells. This hypothesis is also supported by other work where sequencing analysis of the VP1 gene and/or non-coding control region (NCCR) in samples obtained from PML patients showed a similar dichotomy between urine on the one hand and plasma and cerebrospinal fluid (CSF) on the other hand [37–39]. Whether crosstalk between both viral propagation zones exists, is unclear and remains to be investigated.

We found that increased viral multiplication in the urinary propagation zone, as determined by increased urinary viral load, is accompanied by an increased level of urinary viral miRNAs. This might at first sight appear in contradiction with the observation that polyomavirus miRNAs, including JCPyV, have been shown to downregulate expression of large T-antigen and to suppress viral replication [28, 40–42]. One might therefore assume that an increased level of viral miRNAs would be associated to a lower viral load. This mechanistic model does however not exclude that in subjects with high urinary viral load, viral miRNAs are released at increased levels as a consequence of high virion production, a process that requires transcription of the late transcript, which also encodes the miRNAs. Furthermore, several studies have shown that infectivity of mutant viruses lacking the viral miRNAs are not impacted, again indicating that increased levels of viral miRNAs are not per se associated with decreased virion production *in vivo* [28, 43]. On the other hand, these miRNAs can also be produced and released from an infected cell without the need for active viral replication, as is also the case for Herpes Simplex Virus and Epstein-Barr Virus during latent infection [44–46]. This would in fact also explain why no viral DNA can be detected in plasma of healthy subjects, while viral miRNAs are detectable in a large number of these subjects. Taken together, low levels of circulating viral miRNAs would serve as biomarker for latent JCPyV infection and increased levels might be indicative of increased viral activity. This raises the question whether an increased viral multiplication in the bone-blood-brain propagation zone, as is the case in PML patients, also is accompanied by an increase in plasma viral miRNAs. Therefore, it would be of great interest to determine miRNA levels in plasma of PML patients, but even more to assess in longitudinal studies whether plasma miRNA levels in MS patients treated with natalizumab increase over time and as such might serve as a monitoring tool for viral reactivation. Similar studies have been performed for the closely related BKPyV in the context of kidney transplant patients developing polyomavirus-associated nephropathy (PVAN) and found strongly increased levels of miR-B1-5p in urine from patients with PVAN [42]. Also, a strong correlation appears to exist between BKPyV encoded miRNAs and BKPyV DNA in blood of infected renal transplant patients [47].

Since the three miRNAs analyzed in this study are very similar, the cross-reactivity of the different assays was evaluated using synthetic miRNAs. Although we believe that we have provided sufficient evidence that the detected levels of JCPyV miRNAs are specific for JCPyV, it cannot be excluded that the assays used are prone to some non-specific amplification of other miRNAs. However, no miRNAs with nucleotide sequence resembling the sequence of jcv-miR-J1-5p have been registered in miRBase 21, except for bkv-miR-B1-5p [48]. It can also not be excluded that other, unknown human polyomaviruses exist that encode an identical or closely related miRNA that is detected in our assays.

## Conclusions

Based on the work described here, we conclude that JCPyV miRNAs can be detected in plasma and urine of healthy subjects, allowing further stratification of seropositive individuals. Furthermore, our data indicate higher infection rates than would be expected from serology alone.

## Materials and methods

### Ethics statement

The Ethics Committee [“Commissie voor Medische Ethiek - ZiekenhuisNetwerk Antwerpen (ZNA) and the Ethics committee University Hospital Antwerp] approved the Protocol, and Informed consent, which were signed by all subjects.

### Healthy subject samples

A total of 50 healthy subjects (HS) were recruited in Belgium for this study [10, 26, 49]. The demographic description of the HS population is presented in Table 1. Plasma samples and urine samples were collected from all these HS and stored at -80°C until further processing.

### JC polyomavirus viral load assay

Analysis of the urinary viral load was performed as described previously [10]. Analysis of the plasma viral load was performed similarly, with the exception that 200 μL plasma was used for DNA extraction.

### JC polyomavirus VP1 serology assay

The anti-JCPyV antibody assay was performed as described earlier [26]. Samples were considered positive if OD values were higher than 2-fold the OD value of the blank sample (i.e. log_2_ test/ctrl > 1).

### Synthetic microRNA molecules and generation of miRNA standard curves

Three RNase-free 5’-phosphorylated miRNA oligoribonucleotides were synthesized (Integrated DNA Technologies) for the validation of the miRNA assays, corresponding to jcv-miR-J1-5p (5’-phospho-UUCUGAGACCUGGGAAAAGCAU-OH-3’), jcv-miR-J1a-5p (5’-phospho-UUCUGAGACCUGGGAAGAGCAU-OH-3’) and bkv-miR-B1-5p (5’-phospho-AUCUGAGACUUGGGAAGAGCAU-OH-3’). Stock solutions of 100 μM synthetic oligonucleotide in RNase-free and DNase-free water were prepared according to the concentrations and sample purity quoted by the manufacturer (based on spectrophotometric analysis). These stock solutions were diluted to a concentration of approximately 3.32 pM, corresponding to 2.10^5^ copies/μL. A total of six serial 10-fold dilutions were prepared, starting from 2.10^5^ copies/μL down to 2 copies/μL and additional no template controls (NTC; zero copies) were examined. Dilution series of each of the synthetic miRNAs were made in RNase-free and DNase-free water.

### Analysis of viral miRNAs

Levels of circulating viral miRNAs were determined by means of quantitative reverse transcriptase PCR (qRT-PCR) with hydrolysis probe based miRNA assays, purchased from Life Technologies. Specific assays were used for jcv-miR-J1-5p (Assay ID 007464_mat), jcv-miR-J1a-5p (Custom Assay ID CS39QVQ) and bkv-miR-B1-5p (Assay ID 007796_mat). As extraction and reverse transcription efficacy control, assays specific for human miRNAs hsa-miR-26a (assay ID 000405), hsa-miR-30b (assay ID 002129) and mmu-miR-93 (assay ID 001090) were included in the analysis.

Briefly, RNA was isolated from 200 μL plasma or urine using the miRNeasy kit (Qiagen) according to the manufacturer’s instructions. Three μL of total RNA (representing 20% of total RNA extract) or synthetic miRNA solution was reverse transcribed using the pooled RT stem-loop primers (Life Technologies), enabling miRNA specific cDNA synthesis. Subsequently, 2.5 μL of the RT product (representing 1/6 of total RT product) was pre-amplified by means of a 12-cycle PCR reaction with a pool of miRNA specific forward primer and universal reverse primer to increase detection sensitivity. Diluted (1:8) pre-amplified miRNA cDNA was then used as input for a 45-cycle qPCR reaction with miRNA specific hydrolysis probes and primers (Life Technologies). For analysis of the viral miRNAs, 2 μL of input was used. For analysis of the human miRNAs, 2 μL of input was used for urine derived miRNAs and 0.2 μL was used for plasma derived miRNAs. All reactions were performed in duplicate on the LightCycler® 480 instrument (Roche Applied Science). Quantification cycle (Cq) values were calculated using the 2^nd^ Derivative method with a detection cut-off of 40 cycles. Only samples with quantifiable Cq values for both duplicates were considered positive. Absolute miRNA levels in plasma or urine were calculated based on the standard curves for each specific miRNA assay. Limit of quantification (LOQ) was defined as the miRNA concentration corresponding to a Cq value of 40, based on the standard curve for the specific miRNA. For each sample, average Cq value of the 3 human control miRNAs was calculated and possible outliers were identified using Grubbs’ test (using a significance level of 0.05). In case outliers were detected, results of the corresponding viral miRNAs were not included for further analysis.

### Statistical analysis

Differences in relative occurrence of viral miRNAs between groups were assessed using a Fisher’s test. Differences between groups were considered statistically significant at *P* < 0.05. Differences in miRNA levels between groups were assessed using a Mann-Whitney test. Differences between groups were considered statistically significant at *P* < 0.05. Correlation between different parameters was analyzed using linear regression. *P*-value was calculated to determine whether slope was significantly non-zero and strength of correlation was determined using r-value. All statistical analyses were performed using GraphPad Prism v 5.04.

## Electronic supplementary material

Additional file 1: Table S1: Calculated contributions of non-specific detection. Based on the calibration curves for each specific assay, the concentration of the specific miRNA in every sample is calculated. Based on that concentration and the calibration curves for the non-specific assays, a calculated Cq value is then determined for each of the other assays. In case this calculated Cq value is higher than 40, it is assumed that there is no contribution of non-specific detection of that particular miRNA in that particular assay. This approach is also visually presented in Additional file 2: Figure S1. (ZIP 28 KB)

Additional file 2: Figure S1: Calculation of the contribution of non-specific detection. Based on the standard curves the contribution of non-specific detection of a miRNA on the Cq value of another miRNA assay is calculated. First, based on the Cq value obtained from the specific assay and its specific standard curve, the miRNA level is quantified (indicated by “1”). Subsequently, it is calculated what the Cq value would be in the non-specific assays using extrapolation of the non-specific standard curves (indicated by “2”). (PNG 215 KB)

Additional file 3: Table S2: Individual results of JCPyV VP1 antibody levels, urinary viral load and plasma viral load. (ZIP 12 KB)

## Abbreviations

PML: Progressive Multifocal Leukoencephalopathy
JCPyV: JC Polyomavirus
miRNA: microRNA
HSs: Healthy subjects
qPCR: Quantitative polymerase chain reaction.
