# Supplementary figures and images for: Viral miRNAs in plasma and urine divulge JC polyomavirus infection
Source: Virol J. 2014 Sep 2;11:158. doi: 10.1186/1743-422X-11-158 (PMC4168162; doi:10.1186/1743-422X-11-158)

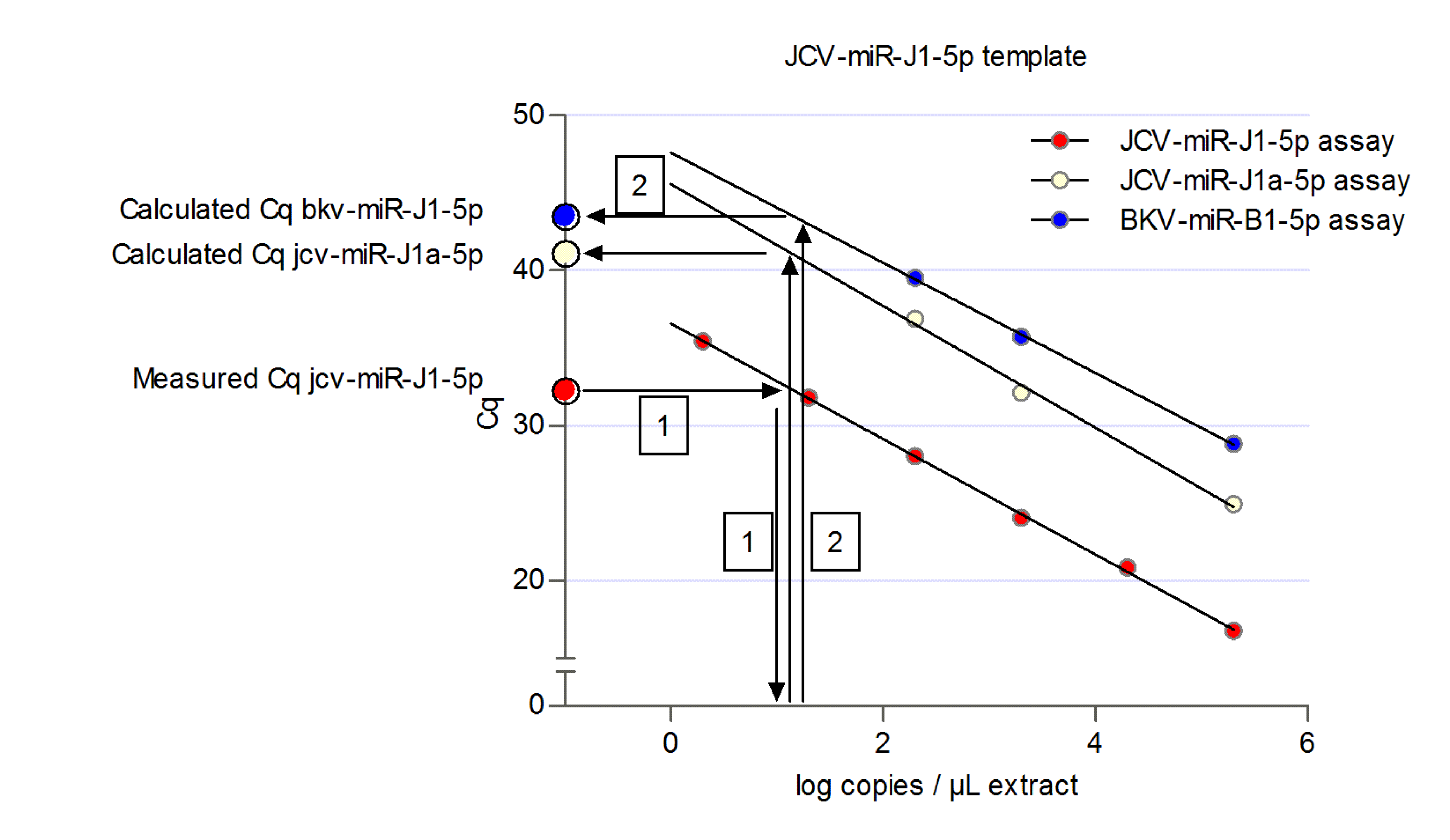

Supplement: Supplementary file 2 — Additional file 2: Figure S1: Calculation of the contribution of non-specific detection. Based on the standard curves the contribution of non-specific detection of a miRNA on the Cq value of another miRNA assay is calculated. First, based on the Cq value obtained from the specific assay and its specific standard curve, the miRNA level is quantified (indicated by “1”). Subsequently, it is calculated what the Cq value would be in the non-specific assays using extrapolation of the non-specific standard curves (indicated by “2”). (PNG 215 KB) [file 12985_2014_2490_MOESM2_ESM.png]
